# Reports of Societies

**Published:** 1875-05

**Authors:** 


### Reports of Societies.

### CHICAGO SOCIETY OF PHYSICIANS AND SURGEONS.

(Reported by RALPH E. STARKWEATHER, M.D.)

Regular meeting April 26, 1875. The President, Dr. John Bartlett, in the chair.

Dr. Plym. S. Hayes read the following paper:

*Experimental Observations on Salicylic Acid.* By PLYM. S. HAYES, M.D.

It may not be without interest to review some of the considerations which caused the following experiments to be made.

In the article on Salicylic Acid that Dr. Squibb read before the Medical Society of New York, there occurs the following: "It is said to reach and prevent processes of decomposition which are beyond the reach of all other antiseptics or antiferments." This, in connection with the suggestion of its internal use, and the following from Dalton's Physiology: "The action which is exerted upon the food by the digestive fluids is not that of a simple chemical solution. It is a transformation, by which the ingredients of the food are altered in character at the same time that they undergo the process of liquefaction. The active agent in producing this change is in every instance an organic matter, which enters as an ingredient into the digestive fluid; and which, by coming in contact with the food, exerts upon it a *catalytic action*, and transforms its ingredients into other substances;"—were the means of framing the question, Would the administration of salicylic acid at or near the time of the ingestion of food arrest or retard in any manner the action of the various digestive ferments, such as the saliva, gastric juice, etc., on the ingested food? It might be well, just here, to explain the reason why the word catalysis, as used by Dalton, was replaced by ferment. Catalysis in the older chemistries occupied a place of great importance, and was the pack-horse on whose back was strapped many of the then inexplicable phenomena connected with this science. It is almost entirely and in some cases quite ignored in our more recent text books. Fermentation which has in a measure supplanted catalysis, refers in its most restricted sense only to that action; the cause of which is almost entirely due to living organisms. Dr. Squibb calls this class of ferments "vital." That produced by yeast, and many which occur in putrefaction, are examples of this form of fermentation. That chemical action in which living organisms have no influence; but the metamorphosis is caused by the action of some physical agent, or of some definite chemical compound laborated by an organism—some organic substance—the same authority calls "chemical," "those which occur independent of vitality, as the production of volatile oil in mustard and bitter almonds, the effects of diastase," etc. As long as chemistry offers no explanation of "chemical" ferments, and scientific men are content to use the term, and professors in our medical colleges place the action of saliva, gastric juice, etc., under the head of ferments, we, too, may use the term without censure.

In order to experimentally answer the question, some of Squibb's unbleached salicylic acid was procured, which he says "is probably pure enough for almost all, if not for all, the purposes to which the acid is at present applied to practical uses." With this I tried two series of experiments. The first series was instituted to determine if salicylic acid had the power of preventing the formation of glucose when saliva had been added to starch which contained a portion of acid.

The second series was to determine whether the acid arrested or retarded in any measure the action of gastric juice—or its active principle, pepsin—on coagulated albumen.

First Series. The first experiment consisted in the addition of saliva to hydrated starch, with which had been boiled salicylic acid. There was apparently as much sugar present as there would have been had not the acid been employed. Before making the experiment it was observed that the acid had apparently undergone decomposition from some unknown cause. The surface of the starch was covered with iris colored patches, such as are seen when a thin film of oil is spread on the surface of water.

Less sanguine of success in proving that the acid was capable of preventing or modifying the action of saliva on starch, the second experiment was entered upon. This consisted in the addition of salicylic acid to cold hydrated starch. After thoroughly agitating the mixture, saliva was added and the agitation continued for two or three minutes when the test was applied. In this no sugar was discovered.

The third experiment was a repetition of the last one. There was indication of a very minute trace of sugar. This was so slight that even after standing for hours there was hardly a perceptible precipitate. The slight change in color of the mixture was the only indication of sugar.

The fourth experiment was a repetition of the first. In this there appeared to be no decomposition of the acid. The chemical reaction demonstrated that although sugar was present, yet the quantity was not nearly as great as it would have been, provided the acid had been left out.

In the fifth experiment the acid was added to the starch when cold, and the saliva remained in contact with the prepared starch for nearly two hours before the test was applied. The indications of sugar were only slight.

In the sixth experiment a much larger quantity of acid was boiled with the starch than at any previous time. The saliva remained in contact with the starch mixture for two hours. The indications of sugar were only slight. The test used was a modification of Trommer's. The reactions of the above experiments were compared with similar reactions in which the salicylic acid was omitted.

The second series consisted of but the following experiment. Into the following solution: ℞. Wyeth's saccharated pepsinæ porci, gr. ij, acidi muriatici, gtt. ij, aquæ, ℨ ij, five grains of the coagulated white of an egg were placed. At the same time, into a similar amount of a like solution half a grain of salicylic acid and five of coagulated albumen were placed. These were placed in separate vials, subjected to the same conditions, and compared from time to time. At the end of forty-eight hours the albumen in the vial which contained the pepsin solution had disappeared, while that which was in the vial containing the acid weighed two and a half grains, thus having lost just one-half of its weight. This albumen was replaced in the same solution, and at the end of ten days had apparently undergone no change.

After completing these experiments my attention was called to two articles in which mention of the internal use of the acid was made.

In connection with the foregoing experiments, quotations from the articles may not be without interest. In an article on salicylic acid, written by Dr. Boyland, in the Medical and Surgical Reporter, occurs the following: "The acid is now given per os, in doses of ten grains, thrice daily, as an antipyretic in pneumonia, with cold baths containing a portion of the same; also in typhoid fever with the same baths." The Boston Medical and Surgical Journal contains the following information: "Professor Kolbe, to prove the innocuousness of salicylic acid, took, for several consecutive days, half a gramme (seven and a half grains) daily, without the slightest observable unpleasant effect. After an interval of eight days he took, for five consecutive days, one gramme (fifteen and a half grains) daily, and then for two days one and a half grammes (twenty-three grains), in alcohol, each day. The digestion was perfectly normal; no traces of salicylic acid could be found in the urine or fæces. (The test is perchloride of iron, which gives an intense violet color.) At no time was there the slightest discomfort."

"The experiment was repeated by Professor Kolbe and eight of his students, all at the same time. Each took on the first day one gramme, and on the second day one and a quarter grammes of salicylic acid. Not one of them was able to observe the slightest derangement of any organs." In neither of the articles is mention made of the time of administration of the acid; whether near the time of the ingestion of food or not.

Taking all into consideration yet from the foregoing experiments we may draw this conclusion, that it would hardly be proper to administer salicylic acid at or near the time of eating. In some cases it might give rise to severe trouble, particularly in those patients whose power of digestion has been enfeebled.

Dr. Squibb states that, "When applied to wounds, it appears immediately in the urine." This he gleans from Thiersch Pharm. Centrahalle.

Professor Kolbe says, when given internally "no trace of salicylic acid could be found in the urine or fæces." If both statements are correct we may look for different effects according as it is administered. If administered by applying it to an abraded surface, it, by passing through the system unchanged, may destroy septic materials already existing in the blood; while such would not be its effect when given by the mouth, if Professor Kolbe's statement is correct, for it would beyond all probability undergo chemical decomposition by the action of the various digestive fluids on it before it could be absorbed.

I added salicylic acid to the fluid of ascites which was rich in albumen and contained blood globules, pus corpuscles and epithelial scales. The acid at once became white, and after standing for several hours there formed on the surface of the fluid a white coagulum, and the whole of the fluid rendered more gelatinous than a sample of the same fluid which was not treated with the acid. The coagulum was removed and washed. It was then dissolved in liquor potassæ and coagulated by the addition of nitric acid, and again dissolved by the potassa.

The fluid up to the time of treating it with potassa was alkaline.

A portion of acid was added to the albumen of an egg mixed with water. A change was at once observed to take place—the acid becoming white—and in the course of a few hours the whole was pervaded by the coagulum.

From these experiments we may conclude, first: that salicylic acid retards digestion in a marked degree when performed artificially; and second, that it slowly coagulates albumen.

### CANCER OF THE STOMACH.

Dr. Etheridge gave the clinical history and treatment of this case, and was assisted by Dr. I. N. Danforth who had made the post mortem examination, and displayed by means of the microscope, to the Society, numerous pathological specimens taken from this case.

The paper has been reserved for publication.

The discussion which ensued called forth, among other facts, the noticeable one that the patient with other members of her family had, thirty years previously, partaken of a broiled chicken which had been kept in a new refrigerator. On the day next following the meal, the entire family was prostrated by acute arsenical poisoning.

From that time forwards the patient had been subject in summer to bilious colic, and had been obliged to refrain from certain articles of food.

Dr. Danforth thought that there had at first been simple inflammation of the pyloric opening of the stomach, and that the tissues inflamed at this point had later in life become invaded by true cancer cells. Epithelial cells, provoked from their regular growth, may become atypical, and so, after years of constant irritation, become malignant; or, epigrammatically, *the lawlessness of growth is the essence of malignancy*, speaking histologically.

QUINIA AND ITS ALKALOIDS.

Mr. Albert E. Ebert, lately the President of the American Pharmaceutical Association, addressed the Society briefly as follows:

There are a number of alkaloids in the Peruvian bark, quinia being the best known and most largely employed. There are besides: 1, quinidia; 2, cinchonidia; and 3, cinchonia. The cinchonia in the red bark is nearly as abundant as quinia.

There has been an immense amount of these "cheaper" alkaloids made while manufacturing quinia, and, there having been no demand for these other alkaloids, they have been stored away, and their cost has been added to that of quinine.

The cheaper alkaloids are said by reputable members of the medical profession to possess the same or nearly the same therapeutical effect as quinine.

The manufacturers have made efforts to introduce these bi-products (the cheaper alkaloids) to the profession and rid their stock of these alkaloids that have accumulated for years while making quinia.

Sweet quinine was then spoken of, and the history of the exposure of the fraud and expulsion of certain parties from the American Pharmaceutical Association.

Cincho-quinine was next referred to.

If the testimonials from physicians in regard to this nostrum are true, then cinchonia sulphate has a large medicinal virtue, for none can be attributed to the quinia it is said to contain.

Mr. Wentzel, of California, a competent and conscientious chemist, has examined this nostrum and found that it contained cinchonia only.

In 1873, Mr. Ebert had himself examined seven different and original samples, microscopically and chemically, and did not find a trace of quinia in them.

In the wholesale market not one of the original packages made before 1874 can be had; they have either been used or bought up.

The samples now sold do not contain the alkaloid quinia but the sulphate salt of quinia, and this only in the *six-tenths* of one per cent.

The success of these alkaloids goes to show that in many respects they are as useful in therapeusis as the quinia sulphate, and as they cost only twenty cents per ounce, are much cheaper.

If extensively used, the price of quinine would be reduced, and the rapid destruction from the forests of these precious trees, would be much lessened.

QUININE BRUTE.

In reply to a question, Mr. Ebert said that the article generally dispensed under the term quinine brute, is chinoidine, or the precipitated extract of cinchona bark, such as is found in the market under the name of "Wetherill's," or "Ellis'" extract of calisaya bark.

The process is published in Parrish's Practical Pharmacy, fourth edition, p. 665.

Trousseau, in his work on Therapeutics, says that quinine brute is a combination of all the cinchona alkaloids in the impure state.

Chinoidine is the residue after the crystallizable principles have been extracted from the mother liquid, evaporated down to a solid extractive consistency. This preparation has the same relation to the crystallizable alkaloids, that molasses has to sugar.

Dr. Etheridge had found that children and others could take quinine agreeably if given in the syrup glycyrrhizæ.

In one instance, a gentleman with intermittent fever, unable to take quinia, was effectually relieved by twenty grain doses of quinine brute.

Dr. Starkweather referred to an article on intermittent fever, by Bouchut, which considered that quinine brute was the most useful remedy to prescribe for children; it is less soluble and bitter, and more easily taken by children. It should be exhibited just after the fever, in the dose of three to six grains daily; this is nearly double the dose of quinine recommended by Bouchut to be given to children, and he adds, that this should be given by enema.

The Society, after transacting considerable and important routine business, adjourned.

